# Focused Ultrasound‐Augmented Delivery of Biodegradable Multifunctional Nanoplatforms for Imaging‐Guided Brain Tumor Treatment

**DOI:** 10.1002/advs.201700474

**Published:** 2018-01-10

**Authors:** Meiying Wu, Wenting Chen, Yu Chen, Haixian Zhang, Chengbo Liu, Zhiting Deng, Zonghai Sheng, Jingqin Chen, Xin Liu, Fei Yan, Hairong Zheng

**Affiliations:** ^1^ Paul C. Lauterbur Research Center for Biomedical Imaging Institute of Biomedical and Health Engineering Shenzhen Institutes of Advanced Technology Chinese Academy of Sciences Shenzhen 518055 P. R. China; ^2^ State Key Laboratory of High Performance Ceramics and Superfine Microstructure Shanghai Institute of Ceramics Chinese Academy of Sciences Shanghai 200050 P. R. China; ^3^ Research Laboratory for Biomedical Optics and Molecular Imaging Institute of Biomedical and Health Engineering Shenzhen Institutes of Advanced Technology Chinese Academy of Sciences Shenzhen 518055 P. R. China

**Keywords:** biodegradable, blood brain barrier, brain tumor, focused ultrasound, theranostic

## Abstract

The blood brain barrier is the main obstacle to delivering diagnostic and therapeutic agents to the diseased sites of brain. It is still of great challenge for the combined use of focused ultrasound (FUS) and theranostic nanotechnology to achieve noninvasive and localized delivery of chemotherapeutic drugs into orthotopic brain tumor. In this work, a unique theranostic nanoplatform for highly efficient photoacoustic imaging**‐**guided chemotherapy of brain tumor both in vitro and in vivo, which is based on the utilization of hollow mesoporous organosilica nanoparticles (HMONs) to integrate ultrasmall Cu_2−_ *_x_*Se particles on the surface and doxorubicin inside the hollow interior, is synthesized. The developed multifunctional theranostic nanosystems exhibit tumor‐triggered programmed destruction due to the reducing microenvironment‐responsive cleavage of disulfide bonds that are incorporated into the framework of HMONs and linked between HMONs and Cu_2−_ *_x_*Se, resulting in tumor‐specific biodegradation and on‐demand drug‐releasing behavior. Such tumor microenvironment‐responsive biodegradable and biocompatible theranostic nanosystems in combination with FUS provide a promising delivery nanoplatform with high performance for orthotopic brain tumor imaging and therapy.

## 1 Introduction

Nanobiotechnology based on versatile organic and inorganic systems has contributed significantly to the fast development of drug delivery nanosystems for efficient cancer diagnosis and treatment. Compared with traditional organic nanosystems, biodegradable inorganic nanoplatforms have attracted widespread attention due to their intrinsic characteristics including multifunctionality, excellent biocompatibility, and relatively high stability in the body fluids, as well as controlled release of therapeutic agents from nanocarriers in the desired sites, especially in the tumor cells.^1^ Glioblastoma multiforme (GBM) is the most common and malignant primary brain tumor with a 5 year survival rate of less than 5%.^2^ Pathologically, glioblastoma diffusely infiltrates normal brain and resides behind a relatively impermeable blood‐brain barrier (BBB), causing the significant challenges for effective neurosurgical management and targeted delivery of chemotherapeutic drugs.^3^ The construction and localized delivery of cancer‐associated, stimuli‐driven biodegradable nanosystems for GBM theranostics (diagnosis and therapy) are of great significance for achieving the desirable theranostic outcome.

Among various inorganic nanosystems, biocompatible mesoporous silica nanoparticles (MSNs) have shown their high performance in molecular imaging,^4^ drug/gene/protein delivery,^5^ and biosensing.^6^ However, their biodegradability is one of the main biosafety issues hindering their further clinical translation. Therefore, great efforts have been devoted to seeking practical strategies to optimize the biodegradation kinetics of MSNs, such as surface modification^7^ and organic–inorganic hybridization to create organic group‐bridged silsesquioxane framework.^8^ Compared with surface‐modification approach, the framework‐hybridization strategy can change the intrinsic framework nature of MSNs, resulting in the specific biological effects and functions in tumor tissue. For instance, it was demonstrated that physiologically active thioether‐bridged MSNs exhibited the unique biodegradable behavior, as well as reducing‐ and ultrasound‐responsive drug‐releasing performance.^9^ In addition, it was revealed that oxamide‐bridged MSNs showed enzymatically biodegradable performance in cancer cells.^10^

Although some newly developed biodegradable MSNs could concurrently achieve the controlled release of anticancer drugs, enhance the therapeutic efficacy and minimize the drug toxicity, there are still two big challenges for them to be successfully applied to GBM treatment. The first obstacle is the selectively permeable BBB, which hampers the transport and diffusion of large molecules (>500 Da) from the vasculature into the brain, thus reducing the effects of chemotherapeutic agents against the malignant brain tumors.^11^ Recently, focused ultrasound (FUS) combined with microbubbles (MB), has been increasingly recognized as a noninvasive strategy to induce transient, reversible, and local BBB disruption.^12^ The biosafety and effectiveness have also been demonstrated by numerous preclinical studies.^13^ The second challenge is the difficulty to achieve the imaging‐guided deep‐seated brain tumor treatment due to the existence of the skull which results in strong light or ultrasound attenuation.^14^ Photoacoustic (PA) imaging, a newly nonionizing imaging technique integrating optical excitation with ultrasound detection, can overcome the depth limits of optical imaging and allow to visualize deep brain tumor with high spatial resolution.^15^ Taking the advantage of exogenous contrast agents with near‐infrared (NIR, 650–900 nm) adsorption, PA imaging contrast can be significantly enhanced.^16^ Among these PA contrast agents, ultrasmall Cu_2−_ *_x_*Se nanoparticles have been recently demonstrated to possess excellent performance in PA imaging effect, long circulation duration, and fast renal clearance capability in subcutaneous tumor model.^17^ However, the application of copper chalcogenides for sensitive PA imaging in orthotopic brain tumor in mice has been rarely studied.

**Figure 1:**
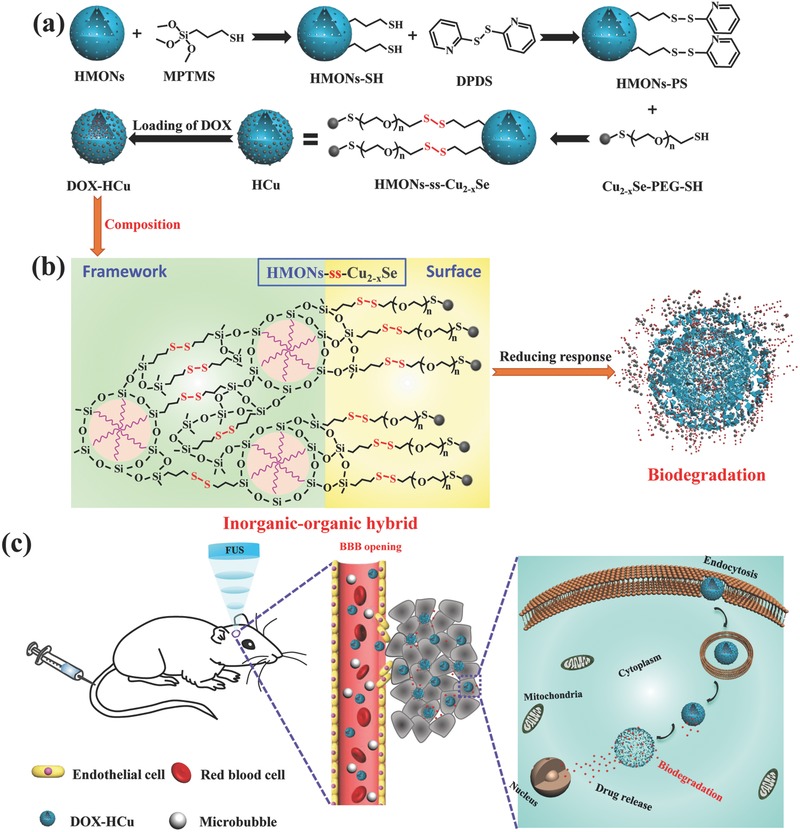
Schematic illustration of a) the synthesis of HMONs‐ss‐Cu_2−_ *_x_*Se (HCu) nanosystems, b) the composition and biodegradable behavior of DOX‐HCu, c) enhanced delivery of DOX‐HCu nanosystems into brain tumor through FUS‐induced BBB opening and subsequent biodegradation of DOX‐HCu caused by the high GSH concentration in the TME.

Herein, we report, for the first time, on the development of novel tumor microenvironment (TME)‐associated smart nanosystems with PA imaging, tumor‐specific biodegradability, and on‐demand drug‐releasing performance for GBM treatment. These nanosystems were constructed by decorating ultrasmall Cu_2−_ *_x_*Se nanoparticles onto the surface of organic–inorganic hybrid hollow mesoporous organosilica nanoparticles (HMONs) via a disulfide linker (HMONs‐ss‐Cu_2−_ *_x_*Se, designated as HCu). The chemotherapeutic drug doxorubicin (DOX) was further encapsulated into the hollow interior of HCu (DOX‐HCu) (**Figure** **1**a). By concurrently triggering FUS exposure, we successfully demonstrated that DOX‐HCu could be accurately delivered into the brain tumor and penetrated into the tumor tissue, thereby resulting in the significant inhibition on tumor progression in an orthotopic brain tumor model (Figure 1c). The as‐synthesized HCu theranostic nanoplatforms possess the following structural and compositional advantages to efficiently diagnose and treat orthotopic brain tumor. First, the ultrasmall Cu_2−_ *_x_*Se nanoparticles on the surface of HMONs not only possess strong NIR adsorption, serving as excellent contrast agents for PA imaging of deep brain tumor, but also have the ability of renal clearance because of the extra‐small particulate size. Second, functional organic group (i.e., disulfide bond)‐bridged silsesquioxane framework of HMONs and disulfide linkers connecting HMONs and Cu_2−_ *_x_*Se are highly active in the reducing environment of tumor tissues, which can improve the biodegradation rate of theranostic nanosystems (Figure 1b). Third, the drug‐loading capacity can be significantly enhanced due to the presence of large hollow interiors of HCu. Especially, the encapsulated drugs exhibit on‐demand drug‐releasing pattern in respond to the reducing environment of TME, resulting in decreased systemic side effects and improved biosafety. Therefore, the concurrent introduction of HCu theranostic nanosystems and FUS‐BBB opening is expected to overcome the impermeable BBB and guarantee the high accumulation of biodegradable theranostic nanosystems into brain tumor tissue, thus achieving the enhanced therapeutic outcome and minimized side effects.

## 2 Results and Discussion

### 2.1 Synthesis and Characterization of HCu Nanosystems

**Figure 2:**
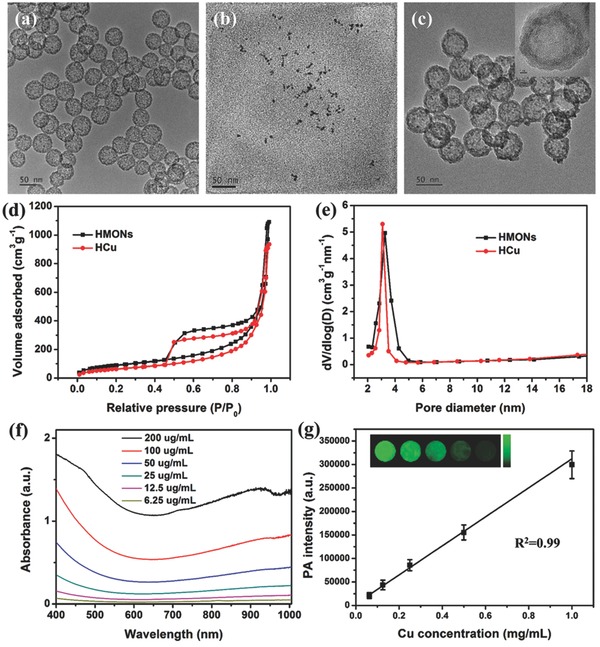
TEM images of a) HMONs, b) Cu_2−_ *_x_*Se, and c) HCu. d) N_2_ adsorption–desorption isotherms and e) corresponding pore‐size distributions of HMONs and HCu. f) UV–vis absorbance spectra of HCu aqueous solutions at different Cu concentrations. g) The linear relationship between PA signal intensity and Cu concentration in HCu aqueous solution. The inset is the PA images of agar gel cylinder at different Cu concentrations.

HMONs were synthesized by employing SiO_2_ as the hard template, bis(3‐triethoxysilylproyl)disulfide (BTDS) with disulfide bond group as organosilica precursor to fabricate SiO_2_@MONs core/shell nanostructure (Figure S1, Supporting Information), followed by etching SiO_2_ core away under alkaline condition. The high dispersity, uniform spherical morphology and hollow structure of HMONs can be clearly observed from the transmission electron microscopy (TEM) image (**Figure** **2**a). The organic group‐hybridized framework and hollow structure are demonstrated by element‐linear mapping (Figure S2, Supporting Information). To obtain the stable theranostic nanosystems (Figure 1a), the as‐synthesized HMONs were first modified with sulfhydryl groups via the typical 3‐mercaptopropyltrimethoxysilane (MPTMS) grafting (HMONs‐SH). Subsequently, ultrasmall Cu_2−_ *_x_*Se‐PEG‐SH nanoparticles (Figure 2b; Figure S3, Supporting Information), which were synthesized according to previous method,^17^ were covalently conjugated onto the surface of HMONs‐SH via disulfide linkers (designated as HCu, Figure 2c). Finally, anticancer drug DOX was encapsulated into the hollow interior of HCu by the typical impregnation process (designated as DOX‐HCu, Figure 1a).

A series of changes in particle sizes determined by dynamic light scattering (DLS) and zeta potential test demonstrate the successful grafting of Cu_2−_ *_x_*Se‐PEG‐SH onto the surface of HMONs (Figure S4, Supporting Information). In addition, the typical N_2_ absorption–desorption technique was employed to characterize the changes of surface area, pore volume, and pore size before and after surface modification of Cu_2−_ *_x_*Se‐PEG‐SH nanoparticles. The results show that HCu nanosystems have a decreased surface area (241 m^2^ g^−1^) and pore volume (1.45 cm^3^ g^−1^) compared with initial HMONs (336 m^2^ g^−1^ and 1.69 cm^3^ g^−1^, respectively) (Figure 2d). Moreover, the initial pore size of HMONs is 3.2 nm, which decreases to 3.0 nm after Cu_2−_ *_x_*Se‐PEG‐SH conjugation, indicating that Cu_2−_ *_x_*Se‐PEG‐SH nanoparticles modified on the surface of HMONs would not block the mesoporous channel (Figure 2e).

The element‐linear mapping (Figure S5a, Supporting Information), energy‐dispersive X‐ray spectroscopy (EDS) spectrum (Figure S5b, Supporting Information) and the color change of nanoparticles (Figure S5c, Supporting Information) further demonstrate the presence of organic group‐bridged framework, hollow structure and anchoring Cu_2−_ *_x_*Se nanoparticles on the surface of HCu. The obtained HCu nanosystems exhibit strong localized surface plasma resonance in NIR region, which is attributed to numerous vacancies arising from copper deficiency.^18^ Moreover, the absorption at 808 nm is linearly increased with the elevated Cu concentration (Figure S6, Supporting Information). Such a strong NIR absorption of HCu nanosystems guarantees their potential application as promising contrast agents for PA imaging, which has been demonstrated by the PA mapping of HCu nanosystems at varied Cu concentrations (inset in Figure 2g). The result shows concentration‐dependent PA signals under 808 nm laser irradiation (Figure 2g).

### 2.2 Stimuli‐Responsive Biodegradation and Drug Releasing

**Figure 3:**
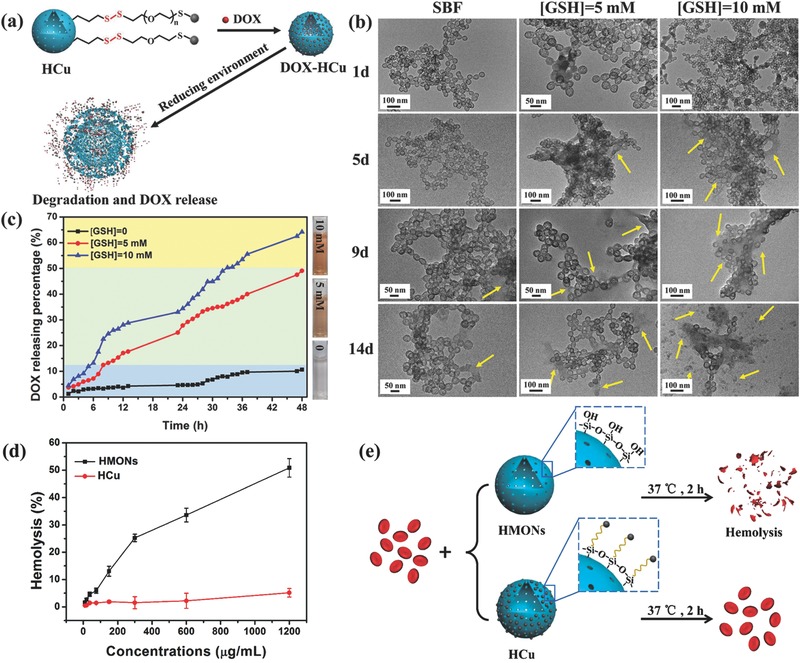
a) Schematic illustration of the degradation and drug‐releasing behavior of DOX‐HCu under reducing environment. b) TEM images of HCu in SBF solution containing different GSH concentrations (0, 5, and 10 × 10^−3^ m) for varied durations (1, 5, 9, and 14 d). c) The releasing profiles of anticancer drug from DOX‐HCu in buffer solutions with varied GSH concentrations (0, 5, and 10 × 10^−3^ m). d) Hemolytic percentage of RBCs incubated with HMONs or HCu at different concentrations. Deionized water and PBS were used as positive and negative controls, respectively. e) Schematic illustration of hemolysis assay procedure and related mechanism.

It has been demonstrated that disulfide bonds are sensitive to the reducing environment,^19^ which have been used to incorporate into the framework of HMONs and to link PA imaging contrast agents (Cu_2−_ *_x_*Se‐PEG‐SH nanoparticles) to HMONs (**Figure** **3**a). To investigate the redox‐responsive biodegradable behavior and drug‐releasing pattern of DOX‐HCu, simulated body fluid (SBF) containing different glutathione (GSH) concentration (0, 5, or 10 × 10^−3^ m) was adopted to mimic the reducing TME, followed by observing the structural evolution of HCu by TEM during the biodegradation process. As shown in Figure 3b, HCu could maintain the relatively intact structure and spherical morphology in pure SBF solution for 14 d. However, substantial changes occur in the microstructure of HCu when they are exposed to the reducing SBF solutions (i.e., in the presence of GSH at elevated concentrations). Notably, structural collapse and incomplete framework of HCu could be more apparent under the higher GSH concentration, indicating the GSH concentration‐dependent biodegradation behavior of HCu.

In the meantime, the DOX‐releasing pattern, as shown in Figure 3c, further reveals that the disulfide bonds within HCu are prone to be cleaved under the reducing conditions. It is shown that the releasing amount of DOX after 48 h is only 10.6% in pure SBF solution, which rises to 49.1% and 64.2% when GSH concentrations increase to 5 and 10 × 10^−3^ m, respectively. These data illustrate that the fast biodegradation of HCu under reducing environment is accompanied by rapid release of anticancer drug encapsulated in the hollow interior. Meanwhile, it is worth noting that Cu_2−_ *_x_*Se‐PEG‐SH nanoparticles decorated on the surface of HMONs would dramatically reduce the number of silanol groups on the surface of HMONs, resulting in decreased hemolytic activity and enhanced hemocompatibility (Figure 3d,e).

### 2.3 Enhanced Intracellular Uptake and Biodegradation of DOX‐HCu

**Figure 4:**
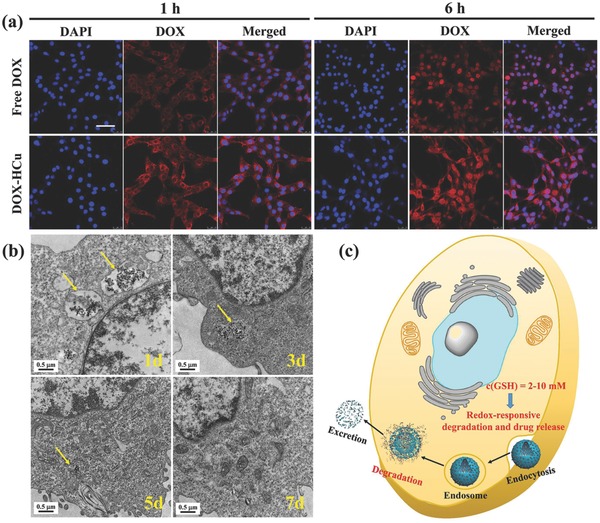
a) CLSM images of U87 glioma cells after coincubation with free DOX and DOX‐HCu for 1 and 6 h ([DOX] = 10 µg mL^−1^, blue fluorescence: DAPI, red fluorescence: DOX). Scale bar = 50 µm. b) Bio‐TEM images for intracellular biodegradation behavior of HCu in U87 glioma cells after coincubation for different durations (1, 3, 5, and 7 d). c) Schematic illustration of intracellular uptake and biodegradation of HCu.

HCu nanosystems reveal no significant cytotoxicity against U87 glioma cells even at a high concentration of 400 µg mL^−1^ in 48 h, indicating their relatively high biocompatibility (Figure S7, Supporting Information). To demonstrate the intracellular drug delivery, U87 glioma cells were preincubated with DOX‐HCu for 1 or 6 h, followed by visualization with confocal laser scanning microscopy (CLSM) (**Figure** **4**a) and quantitative analysis with flow cytometry (Figure S8, Supporting Information). It is found that DOX red fluorescence signals are present in the perinuclear region after 1 h coincubation while in the nucleus after 6 h coincubation, showing a time‐dependent intracellular drug‐delivery manner. Importantly, the DOX fluorescence intensity from DOX‐HCu‐treated cells is higher than that from free DOX‐treated cells (Figure 4a). In addition, flow cytometry analysis reveals that the cellular uptake amounts of DOX‐HCu in U87 glioma cells are time‐ and concentration‐dependent (Figure S8a–c, Supporting Information), indicating that the biodegradation behavior of HCu in the U87 glioma cells can induce larger amounts of DOX release. Significantly enhanced therapeutic efficacy of DOX‐HCu against U87 glioma cells than free DOX was confirmed by determining the cell viabilities after 24 h incubation at varied DOX concentrations (Figure S9, Supporting Information).

Then, we employed bio‐TEM to observe the structural evolution of HCu after coincubation with U87 glioma cells for different durations (1, 3, 5, and 7 d). As shown in Figure 4b, the endocytosed HCu nanoparticles by U87 glioma cells maintain the well‐defined hollow structure and spherical morphology after 1 d coculture. Subsequently, partial nanoparticles are degraded and excreted out of U87 glioma cells. With the prolonged coincubation time, more nanoparticles would be degraded and hardly any nanoparticles could be observable after 7 d coculture. Thus, the high GSH concentration in U87 glioma cells indeed causes the disruption of HCu due to the presence of disulfide bonds (Figure 4c).

### 2.4 In Vivo PA Imaging of Orthotopic Brain Tumor

**Figure 5:**
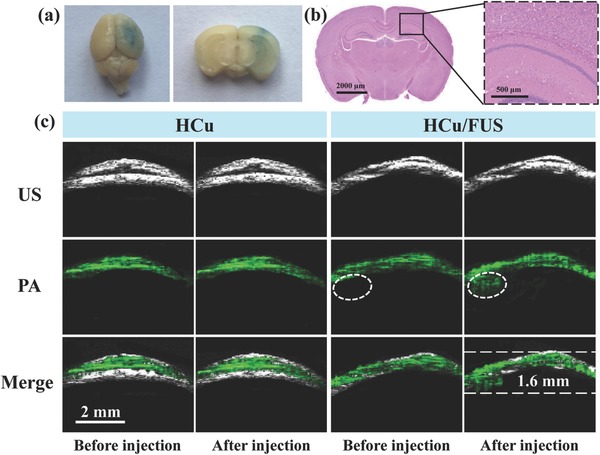
a) EB dye staining of the mouse brain after FUS‐induced BBB opening (Left: an aerial view of a whole brain; Right: brain tissue section). b) Left: The corresponding H&E staining of brain tissue section; Right: Representative microphotograph at high magnification of the boxed area in the left. c) Ultrasound (US), PA, and their overlay images of orthotopic brain tumors acquired before and after intravenous injection of HCu nanosystems without or with FUS‐induced BBB opening.

FUS‐mediated MB destruction has been proven to be a promising approach to induce BBB opening for local delivery of large pharmaceutic agents into the brain.^20^ A successful BBB opening has been achieved by Evans blue (EB) staining of the sonicated brain after exposure to 0.3 MPa acoustic pressure (**Figure** **5**a). No visible brain damage and pathological changes can be observed from the hematoxylin‐eosin (H&E) stained images (Figure 5b), showing a safe delivery of EB dye into the brain. To further evaluate the PA contrast enhancement capability of HCu nanosystems for in vivo deep brain tumor imaging, orthotopic brain tumor model was established by implanting U87 glioma cells into the mouse brain. Prior to the PA experiment, magnetic resonance imaging was employed to accurately position the tumor location (Figure S10, Supporting Information). As shown in Figure 5c, the PA signals (green) before injection of HCu are derived from the blood vessels in the skin and skull.[[qv: 15b,21]] The significant contrast enhancement of PA images is acquired in the tumor beneath the skull after injection of HCu followed by FUS‐induced BBB opening, whereas no obvious PA signals are found in the control without FUS treatment, demonstrating that HCu nanosystems have been successfully delivered into the brain tumor region after BBB opening.

### 2.5 In Vivo Fluorescence Imaging Analysis

**Figure 6:**
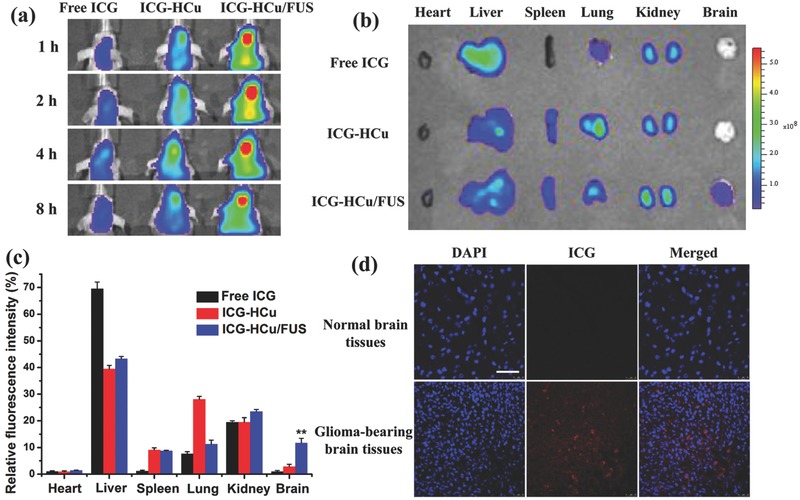
a) In vivo fluorescence images of U87 glioma‐bearing mice after being administrated with free ICG, ICG‐HCu, and ICG‐HCu/FUS at different time points. b) Ex vivo fluorescence images of major organs and brains harvested at 4 h postinjection. c) The quantified relative fluorescence intensity in different organs. **p < 0.01. d) Confocal images of normal brain tissues and ICG‐HCu/FUS treated glioma‐bearing brain tissues. Blue: cell nuclei; Red: ICG‐HCu. Scale bar: 50 µm.

To assess in vivo biodistribution of HCu nanosystems after BBB opening induced by FUS, HCu were fluorescently labeled with near‐infrared dye indocyanine green (ICG) by loading it into the mesoporous channel and hollow interior of HCu. As shown in **Figure** **6**a, the weak fluorescence signal in the brain of ICG‐ or ICG‐HCu‐treated mice is observed after 1, 2, 4, and 8 h intravenous administration, which might be attributed to a few free ICG or ICG‐HCu nanoparticles diffusing through the defective BBB and accumulating in the brain tumor tissue via passive enhanced permeability and retention effect.^22^ Comparatively, a much stronger fluorescence signal in the glioma site is observed in the mice administrated with ICG‐HCu/FUS, suggesting significantly enhanced delivery of ICG‐HCu and deep penetration into glioma tissues after local BBB disruption mediated by FUS. The whole body distribution of HCu was also recorded after intravenous administration (Figure S11, Supporting Information). The ex vivo fluorescence images further demonstrate that the relative fluorescence signal intensity of ICG‐HCu in brain tissue to the total organs is about fourfold higher in the presence of FUS than that in the absence of FUS (Figure 6b,c).

Furthermore, the enhanced ICG‐HCu accumulation in the targeted brain regions was confirmed by CLSM observation. As can be seen from Figure 6d, the significantly strong red fluorescence signals from ICG are clearly observed in the section of ICG‐HCu/FUS‐treated brain tumor tissue. By contrast, scarce fluorescence signal is detected in the normal brain tissue, implying that intravenous injection of ICG‐HCu in conjunction with FUS‐BBB opening has the potential to achieve noninvasive and localized drug delivery for the treatment of orthotopic brain tumor. In addition, the in vivo excretion route after intravenous administration of HCu followed by FUS was also examined (Figure S12, Supporting Information). The results show that the enhanced Si and Cu excretion amounts are found in urine, which are due to the quick biodegradation of HCu and then facile excretion out of the body.

### 2.6 Antiglioma Efficacy

**Figure 7:**
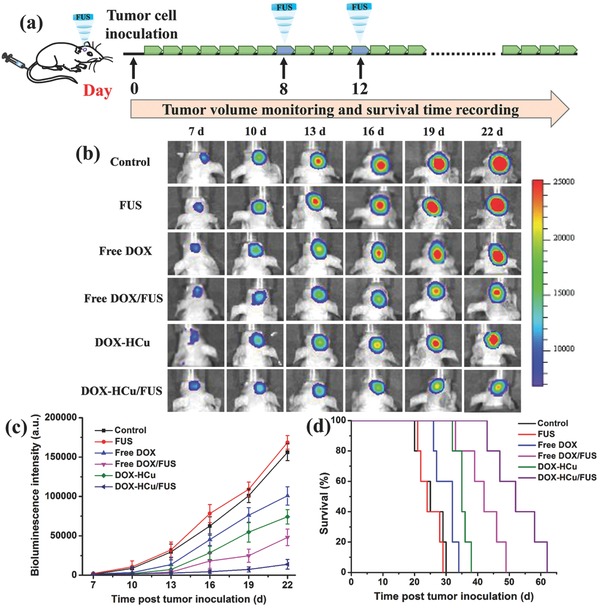
a) In vivo therapeutic scheme of FUS‐mediated chemotherapy in U87‐Luc intracranial brain tumor model. b) The representative bioluminescent images of glioma‐bearing mice from each group. c) The quantitative bioluminescent signal intensity of the mice in all groups. d) Survival curves of glioma‐bearing mice in all groups. Results are mean ± SD (*n* = 5).

To test whether FUS‐mediated drug delivery could increase in vivo therapeutic effect, orthotopic U87‐Luc brain tumor xenograft was established in Balb/c nude mice. The U87 glioma‐bearing mice were randomly divided into six groups: control, FUS alone, free DOX, free DOX/FUS, DOX‐HCu, and DOX‐HCu/FUS groups. The experimental scheme of treatment over time is shown in **Figure** **7**a. As can be seen from Figure 7b,c, similar bioluminescence intensity of brain tumor is observed at day 7 after tumor inoculation in all groups. However, FUS‐mediated DOX‐HCu delivery group exhibits remarkable inhibition of tumor growth (91.1%) compared to control group at day 22, much more effective than free DOX (35.4%), free DOX/US (69.2%), or DOX‐HCu (52.4%) group. It is worth noting that free DOX/US group shows better inhibition efficacy than DOX‐HCu group, illustrating that FUS‐mediated drug delivery is more effective than nanoparticle‐mediated drug delivery in orthotopic brain tumor due to the penetration of chemotherapeutic drugs into the tumor tissue after BBB opening. The median survival time of mice treated with DOX‐HCu/FUS (52 d) is significantly longer than those mice treated with FUS alone (24 d), free DOX (32 d), free DOX/FUS (42 d), and DOX‐HCu (35 d, Figure 7d). The highest antitumor efficiency and longest median survival time achieved by DOX‐HCu/FUS are attributed to the enhanced drug availability at the brain tumor by combined use of HCu nanosystems and FUS irradiation. H&E and terminal deoxynucleotidyl transferase‐mediated dUTP nick end labelin (TUNEL) staining assays reveal significantly enhanced tumor cell damage and apoptosis in DOX‐HCu/FUS group (Figure S13, Supporting Information), confirming its high efficiency in suppressing tumor growth.

### 2.7 In Vivo Toxicity of HCu

**Figure 8:**
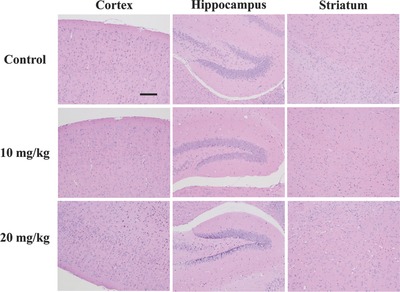
Histopathological examinations of brain tissue from mice after intravenous administration of HCu (*n* = 5, dose: 10 and 20 mg kg^−1^) in combination with FUS. Scale bar: 100 µm.

Although numerous results have demonstrated the low toxicities of MSNs‐based delivery systems,^23^ in vivo toxicity caused by the deposition of nanoparticles in the brain is rarely reported. Here, in vivo histological/hematological biocompatibility of HCu nanoparticles delivered by FUS‐mediated BBB opening was further investigated to guarantee high biosafety for their further clinical translation. No obvious difference in body weight is found between HCu/FUS‐treated groups with two different injection doses and the control group during one‐month feeding (Figure S14, Supporting Information). Then, the tissue sections of cortex, hippocampus, and striatum were obtained to assess the brain toxicity of HCu. As can be seen from **Figure** **8**, no visible tissue damage is observed in the brain after HCu treatment. Meanwhile, there is no noticeable adverse effect or organ damage to heart, liver, spleen, lung, and kidney in the treatment groups (Figure S15, Supporting Information). In addition, blood routine examinations reveal that the blood parameters in the treatment groups fall within normal ranges and have no significant difference in comparison to those in the control group (Figure S16, Supporting Information). All these results evidence that FUS‐mediated HCu nanoparticles are of high biosafety and histo/hemocompatibility.

## 3 Conclusion

In summary, a highly efficient HCu theranostic nanoplatform has been successfully constructed for combating the brain tumor, which is based on the rational integration of organic–inorganic hybrid HMONs with ultrasmall Cu_2−_ *_x_*Se nanoparticles on the surface. Especially, the disulfide bonds incorporated into the framework of HMONs and linked between HMONs and Cu_2−_ *_x_*Se are physiologically active, which can be broken up in the reducing condition of TME, resulting in improved biodegradation and excretion of HCu nanosystems. The rapid biodegradation of HCu could promote anticancer drug release and enhance therapeutic efficacy. Importantly, excellent PA imaging contrast performance and tumor‐inhibition effect by concurrent use of HCu nanosystems and FUS‐BBB opening have been demonstrated in orthotopic brain tumor. Moreover, the obtained HCu nanosystems exhibit lowered hemolysis against red blood cells, negligible systematic toxicities, and high histocompatibility. Therefore, FUS‐mediated HCu delivery nanosystems are expected to provide new insight into TME‐related nanotechnology for the theranostics of orthotopic brain tumor.

## 4 Experimental Section

*Materials*: Tetraethyl orthosilicate (TEOS), triethanolamine (TEA), hydrochloric acid (HCl, 37%), ethanol, and ammonia solution (25–28%) were purchased from Sinopharm Chemical Reagent Co. Cetyltrimethylammonium chloride (CTAC, 25 wt%), MPTMS, 2,2′‐dipyridyl disulfide (DPDS), and glutathione (GSH) were obtained from Sigma‐Aldrich. BTDS was bought from Lark Chemical Technology Co., Ltd. Cell counting Kit‐8 (CCK‐8) and phosphate buffer solution (PBS) was purchased from Beyotime Institute of Biotechnology. All chemicals were used as received without further purification, and their aqueous solutions were prepared using deionized water.

*Preparation of HMONs*: CTAC aqueous solution (2 g, 10 wt%) and TEA aqueous solution (0.08 g, 10 wt%) were mixed and magnetically stirred at 95 °C for 10 min. Then, TEOS (1 mL) was added into the above solution dropwise. After 1 h, the mixture involving TEOS (1 mL) and BTDS (0.6 mL) was added and the reaction was last for another 4 h. After centrifugation, the products were washed with ethanol for several times. Then, the template CTAC was removed by refluxing with a solution of HCl in ethanol (10% v/v) at 78 °C for 12 h. The extraction process was repeated for three times and the products were washed with deionized water for three times and stored in deionized water (20 mL). Finally, sample (5 mL) was diluted in deionized water (100 mL), then ammonia solution (2 mL) was added. The etching process was lasted for 3 h at 95 °C to obtain the final product HMONs.

*Preparation of HCu*: First, HMONs (100 mg) was dispersed in isopropanol (100 mL), followed by adding MPTMS (200 µL) and refluxing at 80 °C overnight. After centrifugation and washing with methanol, the products (HMONs‐SH) were dispersed in methanol (10 mL). DPDS (40 mg) was dispersed in methanol (5 mL), then HMONs‐SH (2 mL) was added and reacted at room temperature overnight to obtain the active HMONs‐PS. Finally, HMONs‐PS was reacted with Cu_2−x_Se‐PEG‐SH to acquire the products HCu.

*Characterization*: TEM images and EDS spectrum were acquired using a JEM‐2100F transmission electron microscope. Element‐linear mapping were captured by FEI Magellan 400 scanning electron microscope (USA). N_2_ adsorption–desorption isotherms were measured on a Micrometitics Tristar 3000 system. UV–vis spectra were conducted on a PerkinElmer Lambda 750 spectrophotometer. DLS measurement and Zeta potential were conducted on a Zetasizer Nanoseries instrument (Nano ZS90).

*Degradation Assay in SBF Solution*: HCu (3 mg) was dissolved in SBF solution (30 mL) with different GSH concentration (0, 5, or 10 × 10^−3^ m). The HMONs SBF solution was magnetically stirred at 37 °C (250 rpm) and a small amount of solution was taken out at the given time. The sample was collected by centrifugation and characterized by TEM observation.

*Drug Loading and In Vitro Drug Releasing under Different GSH Concentrations*: HCu (30 mg) was dispersed in a DOX solution (20 mL PBS, 0.5 mg mL^−1^). DOX‐HCu was obtained after stirring at room temperature under dark condition for 24 h and collected by centrifugation. After washing with PBS for several times, the supernatant was collected and measured by UV–vis spectroscopy at λ = 480 nm to determine the DOX loading efficiency.

For evaluating the drug release behavior, DOX‐HCu (5 mg) was encapsulated into a dialysis bag (molecular weight cut‐off = 5 kDa), and then immersed in SBF solution (25 mL) with different GSH concentration of 0, 5, or 10 × 10^−3^ m. The release procedure was conducted in a shaking table (100 rpm, 37 °C) and the DOX released amount was measured by UV–vis spectroscopy at the given time point.

*Hemolysis Assay*: The red blood cells (RBCs) were obtained by centrifuging the whole blood of Balb/c nude mice and washing with PBS for three times. The diluted RBCs (10% v/v in PBS) were mixed with HMONs under varied concentrations at 37 °C for 2 h. Then the samples were centrifuged and the supernatants were collected. The hemolysis percentage of HCu was determined by measuring the absorbance of the supernatants at 541 nm using UV–vis spectroscopy.

*Cell Culture and Animals*: Human brain glioma cell line U87 cells were cultured in DMEM medium supplemented with 10% fetal bovine serum and 1% penicillin–streptomycin solution in a humidified incubator (5% CO_2_, 37 °C).

Healthy Balb/c nude mice (18–20 g) were purchased from the Medical Experimental Animal Center of Guangdong Province. All animal experiments were performed under the guideline approved by the Animal Study Committee of Shenzhen Institutes of Advanced Technology, Chinese Academy of Sciences. For orthotopic brain tumor model, U87‐Luc cells (5 × 10^5^) in PBS (5 µL) were inoculated into the striatum (bregma 1.8 mm, right lateral 2.0 mm, depth 2.5 mm). The brain tumor growth was monitored by in vivo living imaging system (IVIS Spectrum, PerkinElmer, USA).

*Cellular Uptake*: For CLSM observation, U87 cells were seeded in the confocal dish and cultured overnight, then treated with free DOX and DOX‐HCu (DOX concentration: 10 µg mL^−1^) for 1 and 6 h at 37 °C. At the end of the incubation, the redundant media were removed by washing with PBS for three times. Afterward, the cells were fixed with 4% paraformaldehyde solution and stained by 4′,6‐diamidino‐2‐phenylindole (DAPI), followed by observation under CLSM (Leica TCS SP5). For quantification by flow cytometry analysis, U87 cells were seeded in 24‐well plate at 5 × 10^4^ per well and cultured overnight, then treated with free DOX and DOX‐HCu (DOX concentration: 10 µg mL^−1^) for 1 and 6 h at 37 °C. After addition of 0.4% trypan blue to quench extracellular fluorescence, the cells were harvested and determined by FACSCalibur system.

*Intracellular Degradation Assay*: U87 cells were cultured with HCu (100 µg mL^−1^) at 37 °C for 1, 3, 5, and 7 d. Then the cells were harvested, fixed with 2.5% glutaradehyde for 3 h and 1% osmium tetroxide for 1 h. Followed by dehydration with a graded ethanol solution series, the cells were embedded in Epon by polymerization at 45 °C for 3 h and 65 °C for 48 h. Finally, the ultrathin section was cut and transferred to the copper grid for bio‐TEM observation.

*BBB Opening*: To study FUS‐induced BBB opening, FUS transducer (1.0 MHz and 38 mm diameter) was driven by a function generator connected to a power amplifier. A removable cone filled with degassed water was employed to hold the transducer and guide the FUS beam into the brain. The acoustic parameters were used: acoustic pressure: 0.3 MPa, pulse repetition frequency: 1Hz, duty cycle: 1%, sonication duration 60 s. The microbubbles (mean diameter of about 2 µm and concentration of about 1 × 10^9^ bubbles per mL) were intravenously injected before treatment. To confirm the successful BBB opening, the mice were administrated EB dye (30 mg kg^−1^) via tail veil and sacrificed at 2 h after EB injection. The brain tissue sections were stained with H&E to assess the histological damage.

*In Vivo PA Imaging*: To investigate the in vivo PA imaging performance of FUS‐mediated HCu nanosystems in orthotopic brain tumor, U87 glioma‐bearing mice were anesthetized with 2% isoflurane and placed on a homemade mount. First, the brain was scanned to position the tumor area. Then, HCu nanosystems were intravenously injected with or without FUS treatment. The PA and US images of mice were captured before and 2 h post treatment.

*In Vivo Distribution of ICG‐HCu/FUS in Orthotopic Brain Tumor Model*: U87 glioma‐bearing mice were randomly grouped and intravenously injected with free ICG, ICG‐HCu, and ICG‐HCu/FUS (0.5 mg ICG/kg), respectively. The mice were anesthetized and imaged at 1, 2, 4, and 8 h post administration using the IVIS Spectrum imaging system. For ex vivo fluorescence imaging, the mice were autopsied, and major organs were collected, rinsed, and imaged.

*In Vivo Antiglioma Efficacy*: U87 glioma‐bearing mice were randomly divided into six groups and intravenously injected with saline, FUS, free DOX, free DOX/FUS, DOX‐HCu, and DOX‐HCu/FUS (5 mg DOX/kg), respectively. To monitor the tumor progression, the bioluminescence images were measured at different time intervals after the injection. In addition, the survival time of each group was recorded.

*Histological Analysis*: At the second day post various treatments, tumor tissues of control and DOX‐HCu/FUS groups were harvested, fixed with formalin, and embedded in paraffin. 5 µm sections were cut with a paraffin slicing machine, followed by staining with H&E dyes. Tumor apoptosis was also assessed by terminal deoxynucleotidyl transferase‐mediated dUTP nick end labelin (TUNEL) assay which was carried out with an in situ Cell Death Detection Kit according to the product instruction.

*In Vivo Toxicity Evaluation*: Healthy Balb/c mice (*n* = 5) were administrated with HCu/FUS (10 and 20 mg kg^−1^) through tail vein. The mice with no treatment were used as control. Blood samples and major organs were collected from the control and treated groups at 30 d after injection. In addition, the body weight of mice was recorded every other day for 30 d. HE stained tissue sections were observed under an optical microscope.

## Conflict of Interest

The authors declare no conflict of interest.

## Supporting information

Supplementary
