# Supplementary material for: Focused Ultrasound‐Augmented Delivery of Biodegradable Multifunctional Nanoplatforms for Imaging‐Guided Brain Tumor Treatment
Source: Adv Sci (Weinh). 2018 Jan 10;5(4):1700474. doi: 10.1002/advs.201700474 (PMC5908350; doi:10.1002/advs.201700474)
Supplement: Supplementary file 1 — Supplementary [file ADVS-5-1700474-s001.pdf]

Copyright WILEY-VCH Verlag GmbH & Co. KGaA, 69469 Weinheim, Germany, 2017.

Supporting Information

for *Adv. Sci.,* DOI: 10.1002/advs.201700474

Focused Ultrasound-Augmented Delivery of Biodegradable Multifunctional Nanoplatforms for Imaging-Guided Brain Tumor Treatment

Meiying Wu, Wenting Chen, Yu Chen, Haixian Zhang, Chengbo Liu, Zhiting Deng, Zonghai Sheng, Jingqin Chen, Xin Liu, Fei Yan, and Hairong Zheng**

Copyright WILEY-VCH Verlag GmbH & Co. KGaA, 69469 Weinheim, Germany, 2016.

Supporting Information

Focused Ultrasound-Augmented Delivery of Biodegradable Multifunctional Nanoplatforms for Imaging-Guided Brain Tumor Treatment

Meiying Wu, Wenting Chen, Yu Chen, Haixian Zhang, Chengbo Liu, Zhiting Deng, Zonghai Sheng, Jingqin Chen, Xin Liu, Fei Yan, Hairong Zheng**

Dr. M. Wu, W. Chen, Dr. H. Zhang, Z. Deng, Prof. Z. Sheng, Prof. X. Lin, Prof. F. Yan and Prof. H. Zheng
Paul C. Lauterbur Research Center for Biomedical Imaging, Institute of Biomedical and Health Engineering, Shenzhen Institutes of Advanced Technology, Chinese Academy of Sciences, Shenzhen 518055, P. R. China
Email: fei.yan@siat.ac.cn; hr.zheng@siat.ac.cn

Prof. Y. Chen
State Key Laboratory of High Performance Ceramics and Superfine Microstructure, Shanghai Institute of Ceramics, Chinese Academy of Sciences, Shanghai 200050, P. R. China

Prof. C. Liu and J. Chen
Research Laboratory for Biomedical Optics and Molecular Imaging, Institute of Biomedical and Health Engineering, Shenzhen Institutes of Advanced Technology, Chinese Academy of Sciences, Shenzhen 518055, P. R. China

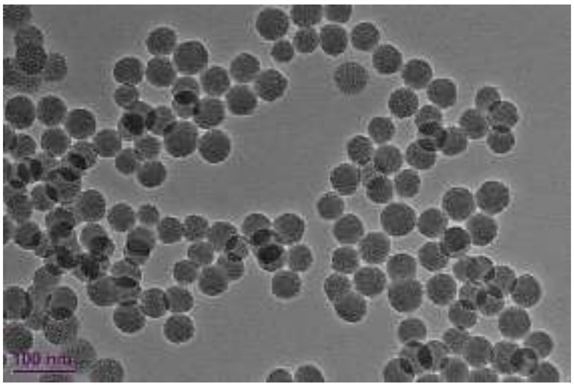

Figure S1. TEM image of SiO_2@MONs.

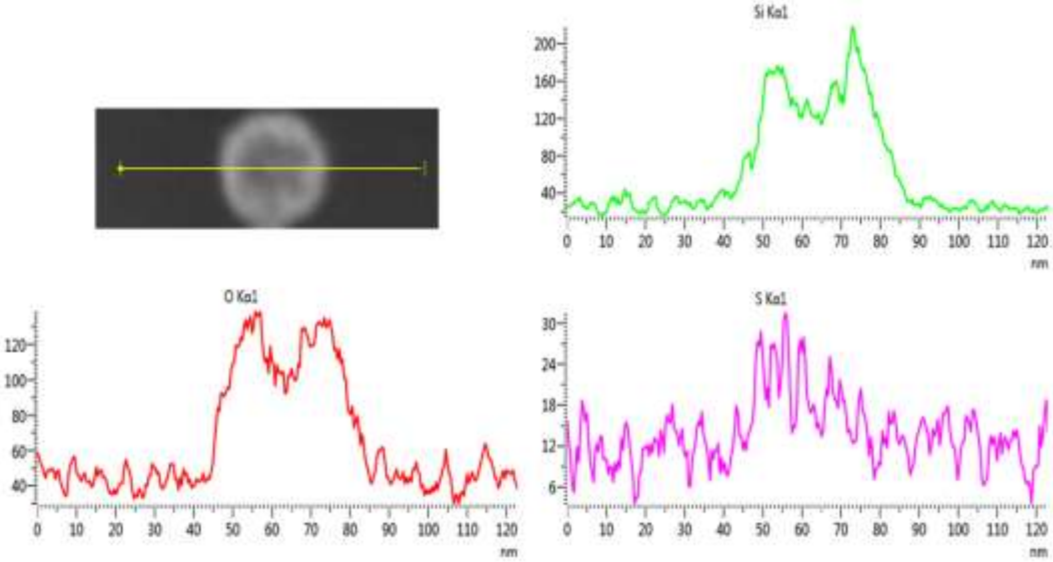

Figure S2. Element-linear mapping of HMONs.

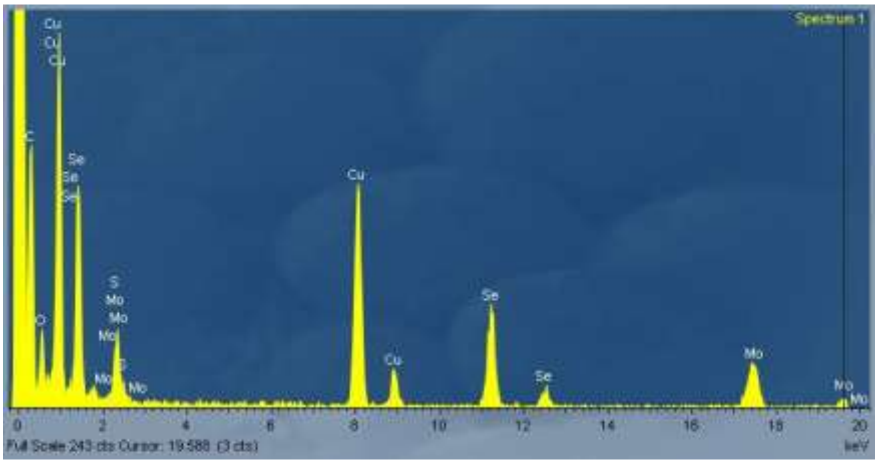

Figure S3. The EDS spectrum of Cu_{2-x}Se-PEG-SH.

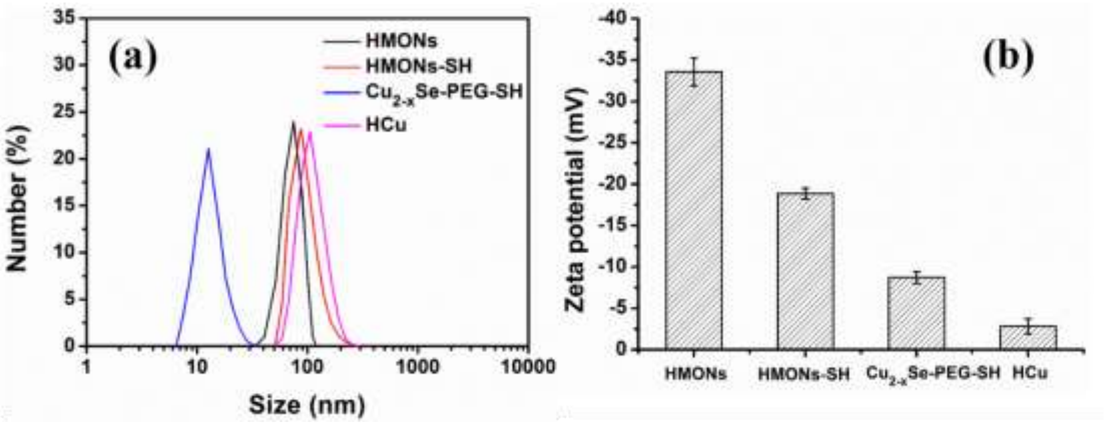

Figure S4. (a) DLS particle-size distributions and (b) zeta potentials of HMONs, HMONs-SH, Cu_{2-x}Se-PEG-SH and HCu.

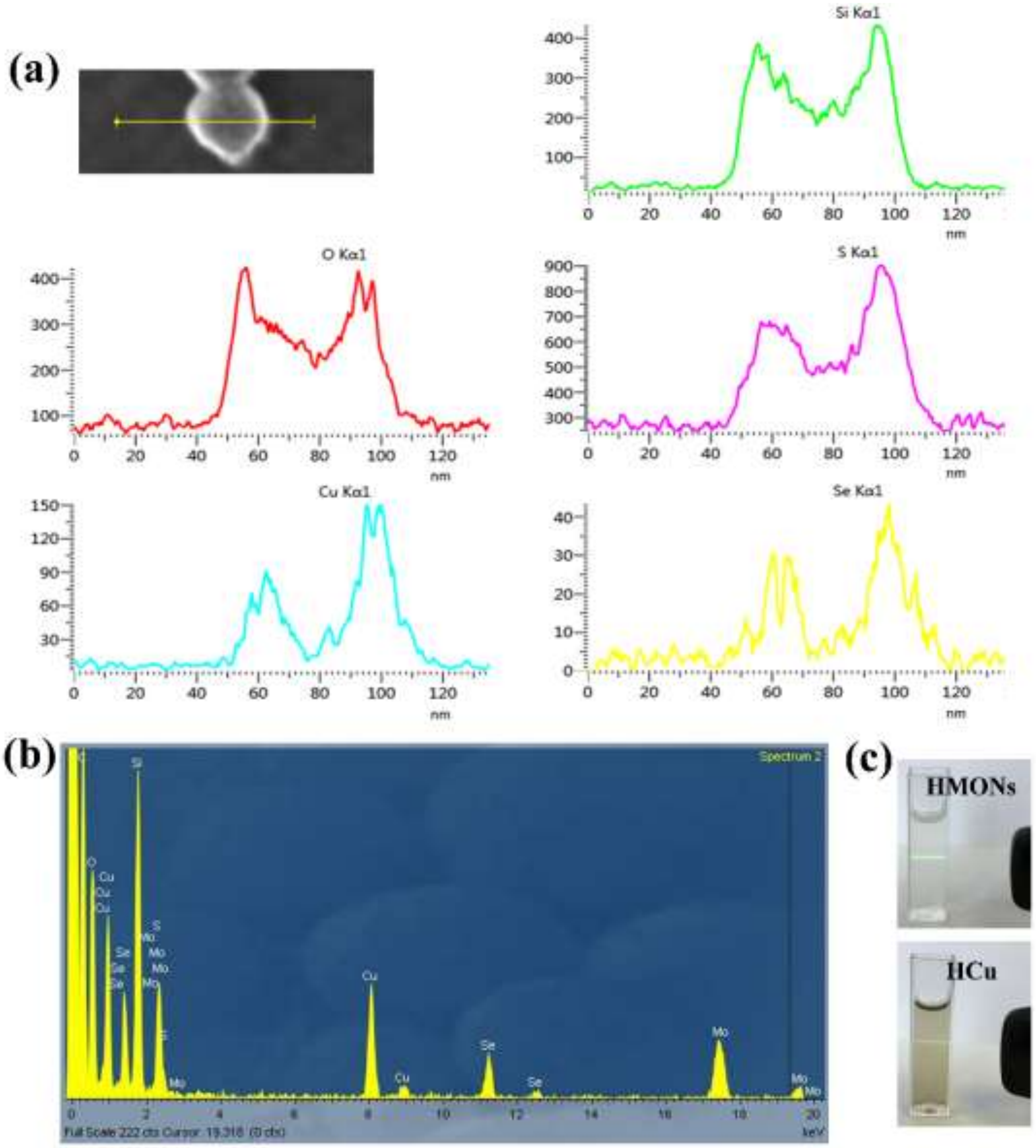

Figure S5. (a) Element-linear mapping and (b) EDS spectrum of HCu. (c) Digital photos of HMONs and HCu well-dispersed in aqueous solution.

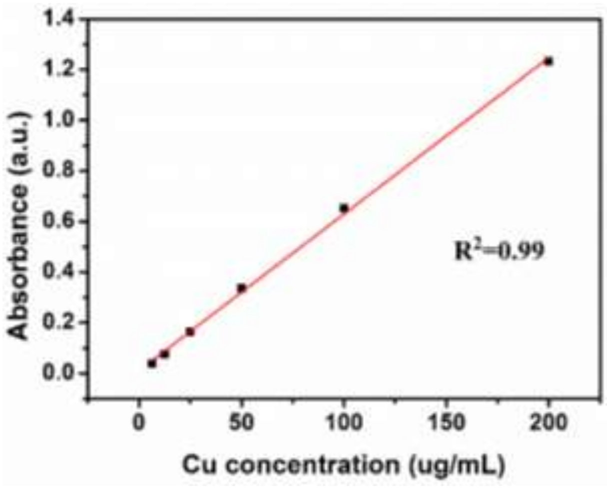

Figure S6. The linear fitting plots of absorbance versus Cu concentration in HCu aqueous solution at 808 nm.

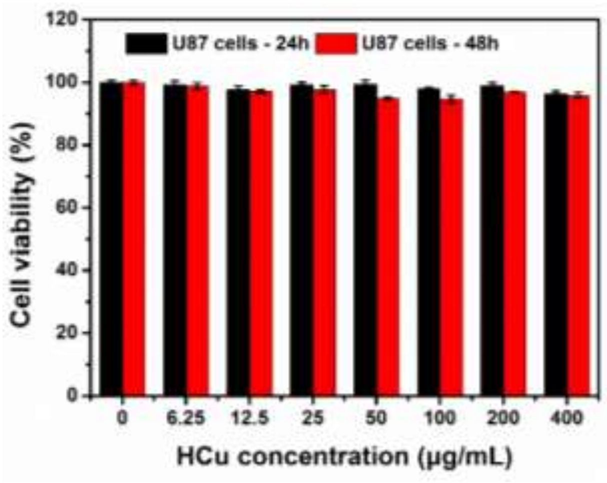

Figure S7. Cell viabilities of HCu against U87 glioma cells at different concentrations in 24 h and 48 h incubation.

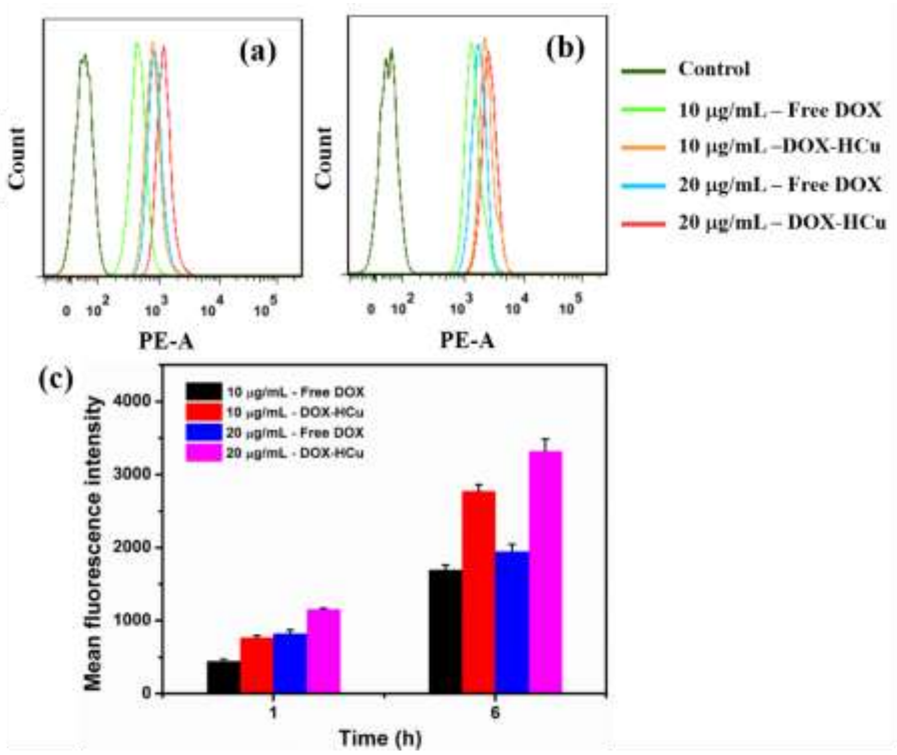

Figure S8. Cellular uptake amounts of free DOX and DOX-HCu in U87 glioma cells in (a) 1 h and (b) 6 h of incubation. (c) The quantified intracellular uptake amounts determined by flow cytometry analysis.

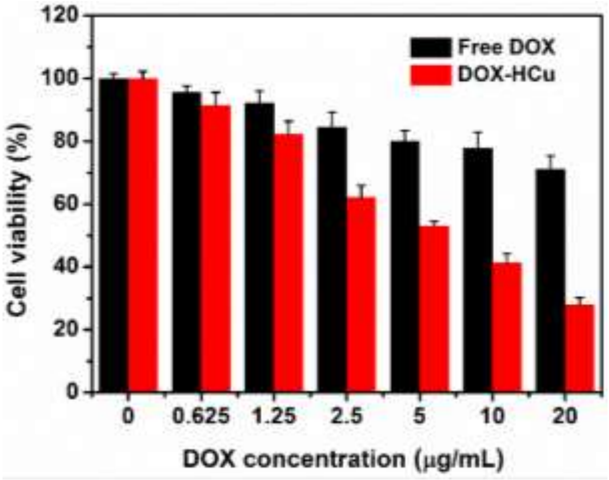

Figure S9. Cell viabilities of free DOX and DOX-HCu against U87 glioma cells at varied DOX concentrations in 24 h incubation.

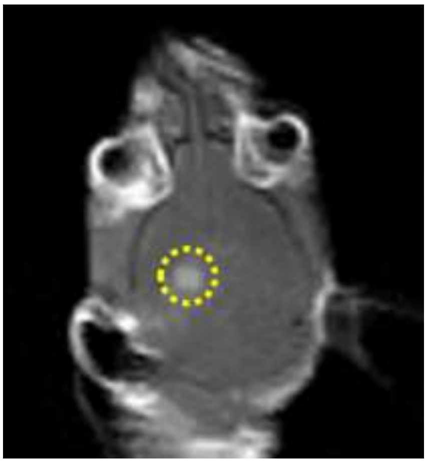

Figure S10. *In vivo* MRI image of the U87 tumor-bearing mouse to confirm tumor location.

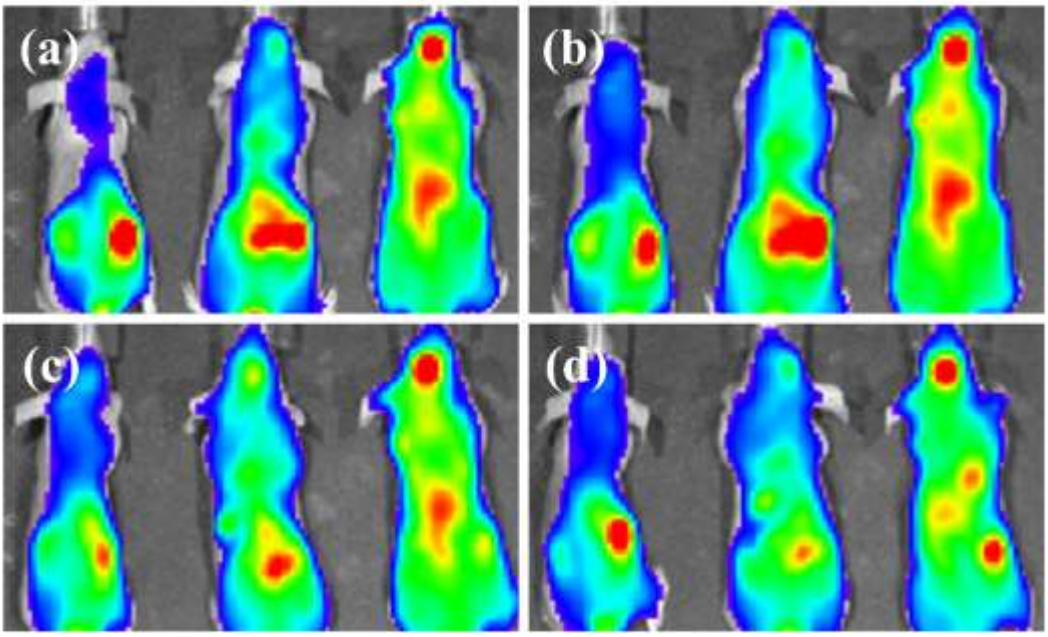

Figure S11. The fluorescence images of the whole body after intravenous injection of free ICG, ICG-HCu and ICG-HCu/FUS at different time points (a: 1 h; b: 2 h; c: 4 h; d: 8 h).

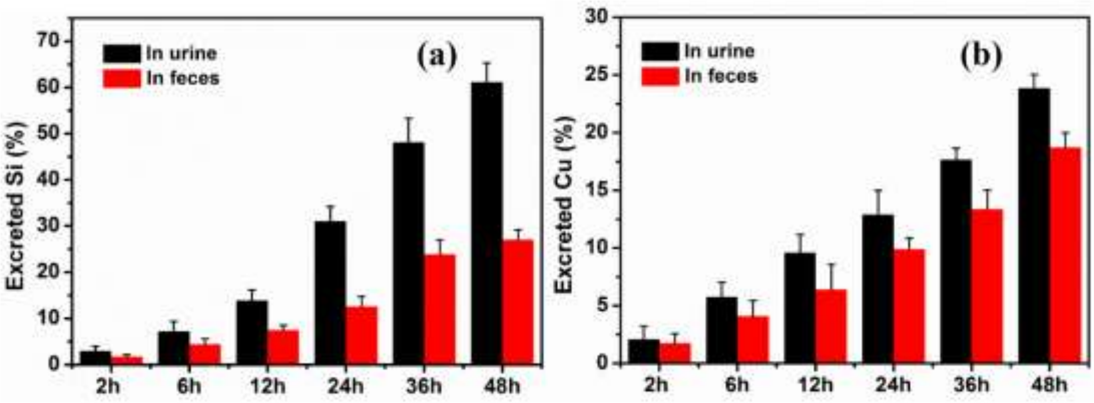

Figure S12. Cumulative (a) Si and (b) Cu excretion in urine and feces of mice after administration of HCu/FUS for varied time durations (2, 6, 12, 24, 36 and 48 h).

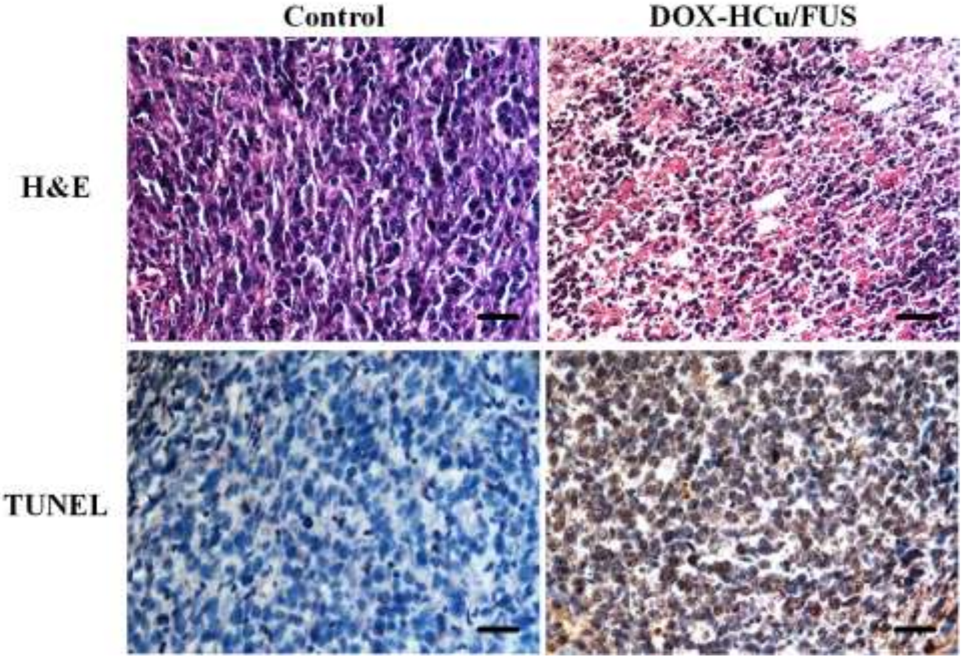

Figure S13. Optical microscopic images of H&E-stained and TUNEL-stained tumor slices collected from control and DOX-HCu/FUS groups of mice 2 d after treatments. Scale bar: 20 μm.

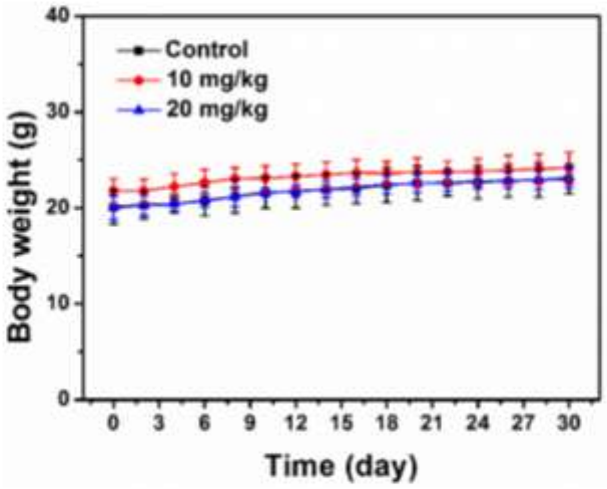

Figure S14. Changes in the body weights of the mice after intravenous administration of HCu (n=5, dose: 10 mg/kg and 20 mg/kg) in combination with FUS.

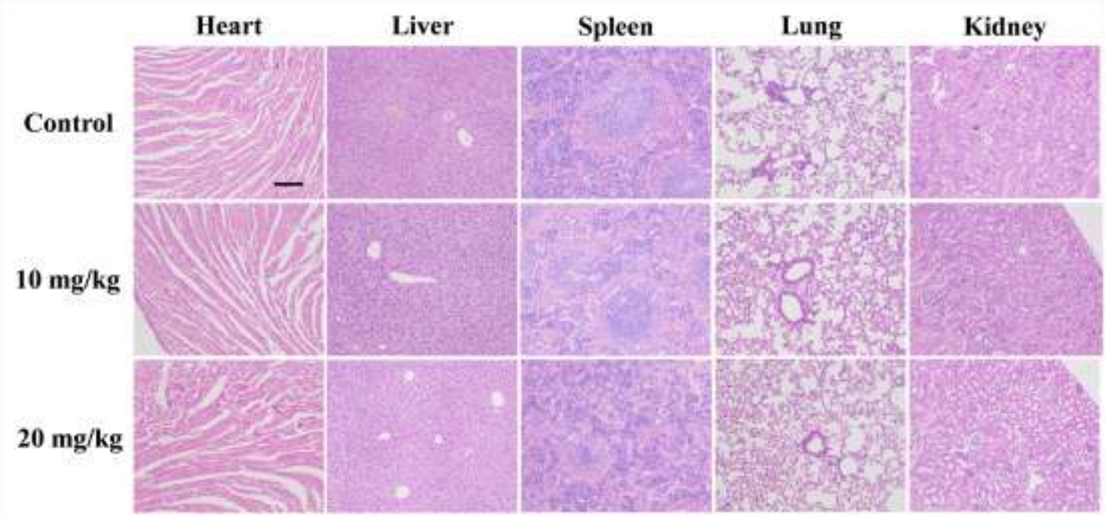

Figure S15. Histopathological examinations of major organs (heart, liver, spleen, lung and kidney) from mice after intravenous administration of HCu (n=5, dose: 10 mg/kg and 20 mg/kg) in combination with FUS. Scale bar: 100 μm.

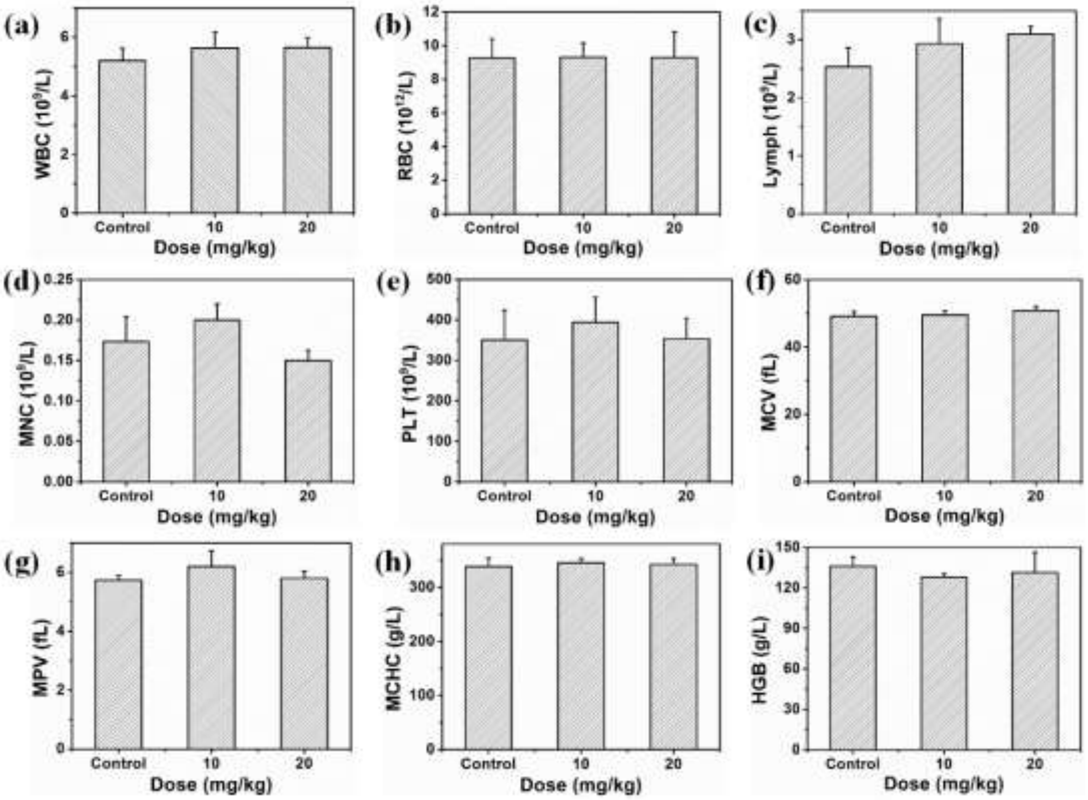

Figure S16. Blood routine examinations of mice after intravenous administration of HCu (n=5, dose: 10 mg/kg and 20 mg/kg) in combination with FUS. (a) number of white blood cells (WBC), (b) number of red blood cells (RBC), (c) number of lymphocyte, (d) number of monocytes, (e) number of platelets (PLT), (f) mean corpuscular volume (MCV), (g) mean platelet volume (MPV), (h) Mean corpuscular hemoglobin concentration (MCHC), (i) hemoglobin concentration (HGB).
